# Vaccine Virus

**Published:** 1854-08

**Authors:** 


*Vaccine Virus.*—We are frequently called upon by our friends from a distance for vaccine virus, but are not always able to furnish it. It will be an accommodation to many, perhaps, to know that it can be obtained of E. L. O'Hara, Druggist, No. 24 West Randolph st., Chicago.

The packages are sent by mail for $1 each. J

